# Assessing the Suitability of a Virtual ‘Pink Warrior’ for Older Breast Cancer Survivors during COVID-19: A Pilot Study

**DOI:** 10.3390/life13020574

**Published:** 2023-02-18

**Authors:** Maria C. Swartz, Michael C. Robertson, Ursela Christopherson, Stephanie J. Wells, Zakkoyya H. Lewis, Jinbing Bai, Michael D. Swartz, H. Colleen Silva, Eloisa Martinez, Elizabeth J. Lyons

**Affiliations:** 1 Department of Pediatrics Research, Division of Pediatrics, The University of Texas MD Anderson Cancer Center, Houston, TX 77030, USA; 2 Department of Nutrition, Metabolism & Rehabilitation Sciences, The University of Texas Medical Branch, Galveston, TX 77550, USA; 3 Department of Kinesiology & Health Promotion, California State Polytechnic University, Pomona, CA 91768, USA; 4 Nell Hodgson Woodruff School of Nursing, Emory University, Atlanta, GA 30322, USA; 5 Department of Biostatistics and Data Science, School of Public Health, The University of Texas Health, Houston, TX 77030, USA; 6 Department of Surgery, The University of Texas Medical Branch, Galveston, TX 77550, USA; 7 Sealy Center on Aging, The University of Texas Medical Branch, Galveston, TX 77550, USA

**Keywords:** physical activity, exergaming, breast neoplasms, physical function, telehealth

## Abstract

The COVID-19 pandemic impacted the conduct of in-person physical activity (PA) interventions among older survivors of BC, who need such interventions to stay active and prevent functional decline. We tested the feasibility of virtually delivering an exergame-based PA intervention to older BC survivors. We enrolled 20 female BC survivors ≥55 years and randomly assigned them to two groups. The intervention group (Pink Warrior 2) received 12 weekly virtual exergame sessions with behavioral coaching, survivorship navigation support, and a Fitbit for self-monitoring. The control group received 12 weekly phone-based survivorship discussion sessions and wore a Mi Band 3. Feasibility was evaluated by rates of recruitment (≥0.92 participants/center/month), retention (≥80%), and group attendance (≥10 sessions), percentage of completed virtual assessments, and number of technology-related issues and adverse events. Intervention acceptability was measured by participants’ ratings on a scale of 1 (strongly disagree) to 5 (strongly agree). The recruitment rate was 1.93. The retention and attendance rates were 90% and 88% (≥10 sessions), respectively. Ninety-six percent completed virtual assessments without an adverse event. Acceptability was high (≥4). The intervention met benchmarks for feasibility. Additional research is needed to further understand the impact of virtually delivered PA interventions on older BC survivors.

## 1. Introduction

The National Cancer Institute considers individuals as survivors of breast cancer (BC) beginning from the time of diagnosis through the end of life [1]. According to this definition, there were 4.1 million survivors of BC living in the United States in 2022 [2]. Although it is encouraging that the survival rate has increased, substantial evidence shows that cancer and cancer treatment can exacerbate age-related declines in physical function [3,4]. Thus, aging survivors of BC are at elevated risk for poor physical function [4] and, consequently, are at higher risk of premature death [5].

Physical activity (PA) has emerged as a key strategy to prevent functional decline and improve quality of life [6]. PA is defined as “any bodily movement that results in energy expenditure” over a period [7]. However, PA engagement in survivors of BC remains suboptimal despite strong evidence of its beneficial effect on physical function, health-related quality of life, and mortality. The prevalence of insufficient PA in breast cancer survivors ranged widely depending on the location and the study population, age, and treatment status, and how PA was measured. For example, a recent study in 2042 women pre and post diagnosed with breast cancer in Germany found that approximately 50% of the participants were insufficiently active pre and post diagnosed, while only 18.2% were sufficiently active [8]. In the United States, a recent study of 1340 female at high risk of developing breast cancer found that one year after treatment, 31.6% were not engaging in recreational/leisure time PA [9].

Adherence to the recommended PA guideline is needed to fully realize the benefits of PA in decreasing symptom burden and improving physical function in BC survivors. Nevertheless, BC survivors reported various perceived barriers to engage in sufficient PA. These include fatigue, pain, limited mobility, and other cancer treatment-related side effects, lack of motivation, time, social support, and limited access to PA programs [10,11,12,13]. Barriers such as lack of motivation, time, social support, and limited access to PA programs are common regardless of disease type [11,14]. However, the added cancer-related symptom burden, such as fatigue, pain, and limited mobility from cancer or cancer treatment, can further impact how BC survivors perceive PA-related barriers and respond to PA interventions [11,12,15,16]. Previous exergame interventions primarily focused on using exergame itself to impact health, but it was not paired with behavioral coaching to enhance self-management skills to overcome PA-related barriers [17,18]. Thus, there is a need to pilot test PA interventions designed to provide both self-management skills to overcome PA-related barriers and enhance survivors’ motivation to engage in PA despite the experience of cancer-related symptoms.

We previously tested the feasibility of promoting PA in an in-person group setting using exergames along with PA behavioral coaching and BC support discussions among survivors of BC aged 18 years and older [19]. PA behavioral coaching was designed to provide self-management skills to overcome PA-related barriers [19]. Exergame was chosen as a tool to promote PA to help reframe PA as a pleasurable activity [20,21]. Accumulating evidence indicates that targeting a person’s motivation and reframing the internal reaction to PA as a fun activity may lead to a more effective intervention [22,23,24,25,26,27]. Our pilot study’s results indicated high levels of acceptance of using exergames and being active in a group setting [19]. Similar to Wurz and colleagues’ findings, feedback from survivors indicated that attending in-person sessions remained a barrier for survivors in all stages of their cancer care continuum (e.g., limited ability to travel because of cancer treatment side effects, traffic) [13]. Thus, there is a need to test the use of videoconference platforms to deliver the intervention to increase participation in group-based exercise, and, in turn, increase physical function capabilities.

The COVID-19 pandemic accelerated advances in videoconferencing technology and made it a more common method of communication via smartphones [28]. Additionally, the COVID-19 pandemic also significantly impacted the conduct and participation of in-person PA interventions targeting medically complex populations, such as older survivors of BC [29]. Thus, we adapted a previously tested exergame- and group-based PA intervention [19] to be delivered via videoconference platform. We also adapted our physical function assessments to be conducted via a videoconference platform [30,31]. The goal was to prevent a decline in physical function among older survivors of BC. Therefore, the overall purpose of this study was to test the feasibility of virtually delivering an exergame- and group-based PA intervention in a sample of older survivors of BC during the COVID-19 pandemic.

## 2. Materials and Methods

**Figure 1:**
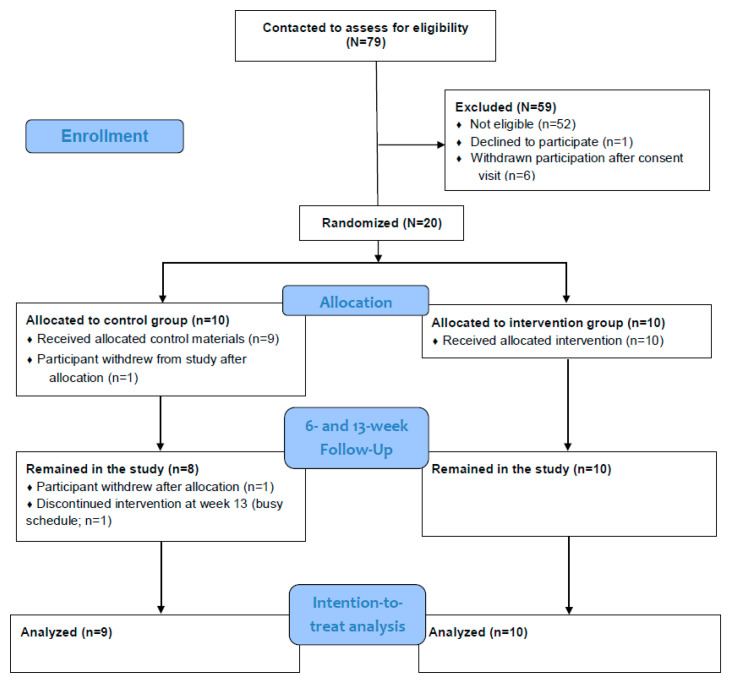
CONSORT pilot and feasibility flow diagram.

We followed the CONSORT (Consolidated Standards of Reporting Trials) 2010 statement for randomized pilot and feasibility trials to report our pilot study (Figure 1) [32]. We conducted a phase II feasibility pilot study [33]. This was a prospective two-group feasibility pilot study in which we used a 1:1 group allocation. The purpose of conducting a randomized pilot and feasibility trial was to increase the likelihood of a successful larger randomized controlled trial by testing the logistics of planned trial [34].

The study aimed to assess the feasibility of a remotely delivered exergame- and group-based PA intervention (Pink Warrior 2) for older survivors of BC. Primarily, the study sought to determine the feasibility of recruitment of the target population, retention, safety of remote physical function assessment, and adherence to the PA intervention. A secondary aim of the study was to explore the potential influence of the intervention on physical function outcomes over 12 weeks.

### 2.1. Participants

Due to COVID-19 pandemic restrictions in clinical settings, participants were recruited from emails and phone calls were drawn from the cancer center’s tumor registry and clinical visit lists between August 2020 and October 2021. An introduction email was sent about the study. Potential participants who expressed interest were contacted by phone to screen for eligibility.

The following were the inclusion criteria to enroll in the study: self-reported female gender, age 55 years or older; having had a primary BC diagnosis; being able to speak, read, and write in English; being able to move arms and legs as well as ambulate; currently being insufficiently inactive (<150 min of moderate-intensity activity per week); having a smartphone, tablet, or computer; and having daily access to reliable internet.

### 2.2. Randomization

Participants were randomly assigned to the intervention group (Pink Warrior 2) or the attention control group. The intervention group received a virtually delivered PA intervention that combined exergame group play, PA behavioral coaching, and BC support (e.g., survivorship guidance). The attention control group participated in weekly telephone and group-based BC support using the BC support discussion materials. The randomization process described by Lyons et al. was used in the current study [19,35]. Briefly, a graduate student who did not assist with the assessments used a web-based app [19,36] to produce a random sequence of treatment assignment to intervention or attention control group using a 1:1 allocation strategy. Each assignment was recorded on a single piece of paper. Then, the graduate student wrapped each treatment assignment paper with a carbon paper and a piece of aluminum foil and then sealed the wrapped assignment in an opaque envelope, which was similar to Swartz et al. [19]. The carbon paper is used to provide an audit trail. The foil was used to ensure that the group assignment was concealed from the assessor before opening the envelope. Lastly, the graduate student shuffled the stack of 20 sealed envelopes and numbered them sequentially by the study ID number and also initialed each envelope to notate the person who had prepared it. The assessor, who was not involved in preparing the envelope, would sign and date the envelope before opening it and save the allocation information in the study file [19].

### 2.3. Procedures

**Figure 2:**
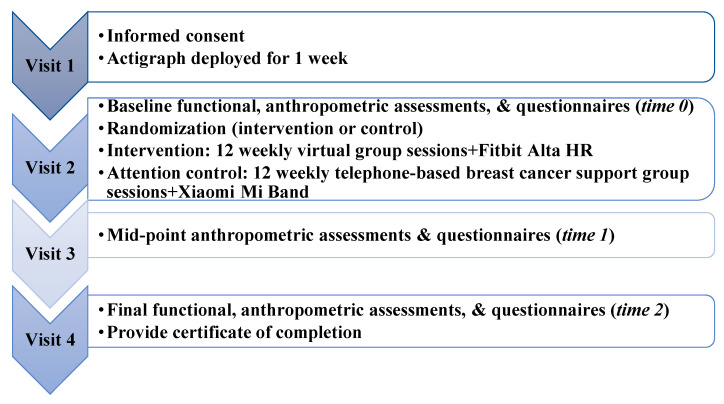
Study flow diagram.

Detailed procedures have been published elsewhere [19]. Briefly, all participants went through four visits virtually for informed consent and assessment. A summary of the study flow is presented in Figure 2. Similar to our in-person design, the study’s duration was 13 weeks, but the Pink Warrior 2 intervention lasted only 12 weeks [19]. Unlike the previous study [19], the current study conducted all study visits virtually using SecureVideo (https://securevideo.com/, accessed on 20 December 2022). The SecureVideo is a HIPAA-compliant telehealth platform. They use the 256-bit AES-encrypted signaling and media stream, and it makes connections to web applications and API through HTTPS only, using TLS 1.3 or 1.2 encryption for in-transit encryption [37]. They also use BitLocker for the 128-bit-AES-encryption for the full database encryption [37]. Additional information on how SecureVideo meets HIPAA standards is provided in the their support center’s About SecureVideo Accounts and Services page [37]. Furthermore, SecureVideo was selected because it included advanced scheduling tools that allowed researchers to pre-schedule the sessions and send automatic reminders to individuals 1-day and 2-h before each session. This feature helped to ensure that participants receive adequate reminders for scheduled virtual visits to promote adherence. Moreover, SecureVideo was reviewed and cleared to be used for the current study by the information security team at the University of Texas Medical Branch. One of the security features provided by the SecureVideo platform was that they provided an individualized unique link through email or text message for each participant to log in to ensure a secure connection and helped the team avoid video-teleconferencing hijacking (also known as Zoom bombing) [38]. Each of the individualized unique links can only be used by one participant. However, the team also turned on the waiting room feature, so only participants who the team recognized could enter into the main room. This was done to further minimize video-teleconferencing hijacking risk. Following the informed consent visit (visit 1), participants were mailed a research-grade activity monitor (ActiGraph GT9X Link accelerometer) to wear for a week as well as baseline questionnaires to complete and return by mail before the baseline assessment visit (visit 2). Participants also provided permission for medical record data extraction as well as an SMS text message, email, or phone call to schedule study visits, and for reminders to be sent prior to assessment and study sessions. The medical record data extraction, SMS text message, email, or phone call were used as additional methods to minimize missed appointments for data collection and promote adherence for attending the group sessions. Additionally, checklists were developed for all study visits, and an Excel spreadsheet was created to track study visits and reminders to follow up on questionnaires and Actigraphs. The research coordinators were trained on the assessments and co-developed the tracking excel spreadsheets. These additional steps were carried out with the intent to minimize missing data.

**Figure 3:**
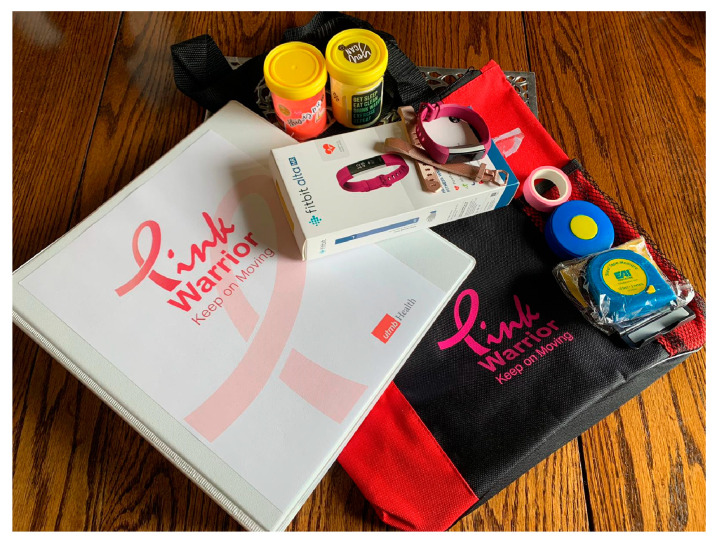
Pink Warrior 2 Assessment and Study Materials.

Using our tracking sheet, we noticed unexpected mailing delays during the COVID-19 pandemic, so the team added additional time between visit 1 and visit 2. After approximately 3 to 4 weeks after visit 1, the study team scheduled visit 2 virtually to conduct a full baseline physical function assessment (time 0). The team also switched to a different courier service (FedEx) to further minimize mailing delays. Participants were randomly assigned to either the intervention group or the attention control group at time 0. All materials were mailed to the participants prior to visit 2 (Figure 3). A study orientation regarding the assigned group along with technology setup was completed at the end of the visit 2. Because of limited staffing resources, we could not conduct a blinded assessment. Furthermore, the study’s design precluded the blinding of patients to their assigned groups. Visit 3 was the assessment halfway through the study (time 1), and visit 4 (time 2) was the final assessment.

### 2.4. Ethics Approval

The Institutional Review Board approved all procedures (protocol 16-0040-02), and our study was registered at ClinicalTrials.gov before study activation (NCT04259905).

### 2.5. Intervention

Detailed description of the intervention has been published elsewhere [19]. Briefly, participants who were assigned to the intervention group took part in a remotely delivered 12-week multicomponent PA intervention. Similar to the in-person design [19], each virtual group session comprised three components: (1) a PA behavioral coaching segment, (2) an exergame-based activity demonstration and practice segment, and (3) a BC support discussion segment. The duration of each weekly structured virtual group session was scheduled to be 60 min. Approximately 15–20 min are spent on the behavioral coaching segment, the exergame-based segment took approximately 30–35 min, and the BC support discussion segment took approximately 10 min.

The Pink Warrior 2 PA behavioral coaching materials were adapted from materials from the Active Living After Cancer (ALAC) program, the details of which were published previously [19,39,40]. Briefly, the Pink Warrior 2 used the same behavioral coaching materials that were developed for the in-person study [19]. The behavioral coaching materials were developed based on the constructs of Social Cognitive Theory and Self-Determination Theory [15,19,23,24,25,26,41]. Under Social Cognitive Theory, we targeted the self-efficacy and self-regulation constructs because both constructs have shown to be associated with the initiation and an increase in PA [22,42]. Under Self-Determination Theory, we specifically targeted the basic psychological needs for autonomy, competence, and relatedness. Self-Determination Theory postulates that by meeting these three basic needs, we would boost the BC survivors’ autonomous motivation, which comes from within an individual, to engage in PA [25]. This would then promote PA over time [22,25,26]. Trained facilitators (UC, a graduate student pursuing a PhD and a licensed occupational therapist specialized in hand therapy and/or MCS, the lead investigator) summarized the weekly PA discussion topics that were designed to provide behavior change skills, which were aimed at promoting the adoption of an active lifestyle. Beyond the group discussion, participants were tasked with completing a weekly reflection worksheet corresponding to the weekly PA coaching discussion topics on their own. The goal of the weekly reflection worksheet was to engage participants to practice using the skills discussed in that week’s PA coaching session and promote an increase in PA outside of the virtual group sessions. Examples of the weekly reflection worksheet included: setting goals, clarifying values related to PA, and finding support for PA.

The exergame sessions involved a facilitator leading the exercise sessions using console-based exergames (e.g., XBOX 360 Kinect). The game selections for the group sessions were previously published [19]. Briefly, the types of games chosen for our in-person and virtually delivered interventions, in collaboration with an occupational therapist, included mind–body games (e.g., Zen energy and yoga games in Your Shape Fitness Evolved 2012), and fitness-based games (e.g., kickboxing, upper and lower-body training in Your Shape Fitness Evolved 2012 and Zumba) [19]. These exergames were chosen as a way to promote enjoyment, increase self-efficacy, and increase motivation to engage in PA in real life [20,43]. Thus, we have selected a variety of activities for participants to choose from that are similar to what they can find in real life or on the web. Each of the exergame sessions consists of a mix of mind–body games and fitness-based games to keep participants engaged. Each game lasted between 5 and 15 min. The length of time for each game depends on the type of game and the level of difficulty. Prior to playing each game, we would explain what the participants can expect, demonstrated the movements used in the game, and demonstrated the type of modifications they can do while playing the game. For example, we demonstrated how participants can use a chair for support when doing the lunge or squat movements.

Unlike the in-person sessions, participants were not provided a game console to use. The exergames were livestreamed via the SecureVideo platform. A similar technique was used in research conducted by Lin [44] that showed that the livestreaming of exergames did not significantly impact participant’s body movement and participation. In our study, the facilitator stood in front of the game console and selected the games based on input from participants. The facilitator’s camera was aimed at the TV monitor and zoomed in on the avatar trainer in the game (e.g., yoga, kickboxing, and Zumba) [19]. Participants then followed the avatar trainer to complete the activity. A YouTube video of the games played during the session (e.g., yoga) was provided to the participants to use throughout the week. However, we also encouraged participants to find other activities they would like to explore beyond the exergame-based activities. The intention was to encourage the adoption of an active lifestyle outside of the group sessions. In addition to the weekly exergame group PA session, participants were provided a Fitbit Alta HR activity tracker. A study email and an anonymous Fitbit account were set up for each of the participants to protect their identity. Participants in the intervention group were able to use the device and the associated Fitbit application (app) to track individual and group steps in a private Fitbit group. This component was incorporated to promote social relatedness [45]. Lastly, we also incorporated hand grip-strengthening exercises with and without TheraPutty. Handouts were developed by UC, an occupational therapist whose specialty is hand therapy. Handouts were provided to the participants to promote engagement in grip exercises throughout the week.

In the BC support segment, resources from the National Coalition for Cancer Survivorship and the American Cancer Society were included to elicit survivorship navigation discussions that were used in our in-person intervention [19]. Briefly, we used the Cancer Survival Toolbox materials from the National Coalition for Cancer Survivorship [46]. Additionally, we used the materials provided in the Personal Health Manager kit from the American Cancer Society. The oncology team we worked with provides the Personal Health Manager kit to all patients when they are first diagnosed with breast cancer [19]. The intent of this component was to equip BC survivors with support and credible resources as they navigate through their cancer experience.

### 2.6. Attention Control Group

Participants assigned to the attention control group (Figure 2) were provided a Xiaomi Mi Band 3 and took part in a weekly telephone-based BC support group for 12 weeks. Attention control was used to ensure the same dose of interaction with a facilitator as intervention participants [47]. A Xiaomi Mi Band 3 was provided as a self-monitoring tool. This activity tracker was selected because the associated app did not include as many behavior change techniques as the Fitbit app [48]. It does provide progressive step goal notifications and a “reach goal” badge when a person reaches the step goal. Similar to the intervention group, a study email and an anonymous Mi Band account were set up for each of the participants to protect their identity. The team also purchased app-based phone numbers for use with the Mi Band app. A master’s degree-level research dietitian (SJW) facilitated the BC support group discussions. The attention control group did not receive the behavioral coaching and exergame components of the Pink Warrior 2 intervention. Instead, they were provided same resources from the National Coalition for Cancer Survivorship and the American Cancer Society as the intervention group to elicit survivorship navigation discussions. Each telephone-based BC support group session also lasted about 1 h. This kind of control intervention was selected because of the well-documented effects of attention in studies promoting behavior change [47].

### 2.7. Primary Outcome Measures

#### 2.7.1. Feasibility

Feasibility was the primary outcome of this pilot trial. Specifically, we evaluated the key elements of the trial, including rates of recruitment, retention, and intervention group attendance and numbers of technology-related issues and participant-reported adverse events. The recruitment rate was defined as the number of participants recruited and randomized per site, per month. The recruitment rate was considered to be feasible if it met the median level of 0.92 participants/center/month [49]. The retention rate was defined as the percentage of participants who completed all endpoint measures. A retention rate was considered to be feasible if 80% or more participants completed the final study assessment [50]. Other aspects of feasibility included the group attendance rate. The benchmark was set at 75% or more participants attending 10 or more sessions for intervention group participants [18,51]. Lastly, we recorded the number of reported technology-related issues and adverse events. The feasibility data were drawn from a database maintenance by the study’s research coordinator.

#### 2.7.2. Acceptability

Similar to our in-person study [19], the acceptability of the virtually delivered exergame- and group-based PA intervention was assessed by using items drawn from Vandelanotte et al. [52,53] and Lyons et al. [35]. The acceptability questionnaire was distributed at time 1 and time 2 (Figure 2). The questionnaire consisted of 11 questions to assess participants’ agreement on a scale of 1 (strongly disagree) to 5 (strongly agree) regarding the use of exergames, the PA behavioral coaching materials, the BC support discussion topics, and satisfaction with the overall program [19]. The program was deemed acceptable if responses were 4 or higher.

### 2.8. Secondary Outcome Measures 

#### 2.8.1. Objective Physical Function Measures

Details of the remotely assessed physical function measures used in the current study are presented in Table 1. The objective physical function measures we conducted remotely included the Short Physical Performance Battery (SPPB) [54], Timed Up & Go (TUG) [55,56,57], and the 2-min step test [58,59]. Handouts providing detailed set-up instructions were provided to the participants ahead of time. The selected objective physical function measures have been previously administered remotely in published studies by Blair et al., Guidarelli et al., and Hoenemeyer et al. [30,31,60]. Blair et al. have been using the 30 s chair stand test, which is similar to the five times sit-to-stand test in SPPB and the TUG test via videoconferencing in older survivors of cancer [31]. The trial has been impacted by the COVID-19 pandemic. Guidarelli et al. tested the SPPB and TUG using videoconference in adults with and without cancer. They found good agreement with in-person tests (ICC = 0.88) for TUG and substantial agreement between repeat assessments for total SPPB score (Cohen’s kappa of 0.78) [30]. Hoenemeyer et al. also found strong agreement (ICC = 0.74) for TUG and very strong agreement (ICC = 0.87) for the 2-min step test among cancer survivors and their partners [60]. Detailed descriptions of how the assessor set up the assessments are included in the Supplemental Material (Table S1).

#### 2.8.2. Physical Activity Measures

The PA metrics used in this pilot study included mean steps per day and mean minutes of moderate to vigorous PA (MVPA) per day. These measurements were obtained using an ActiGraph GT9X Link accelerometer around the waist for 7 days at each time point. ActiGraph is a validated research-grade 3-axis accelerometer. The Troiano algorithm within the ActiLife software was used to estimate wear time and activity. A cut point of 10 h was used to deemed as a valid wear day. Additionally, we used Keadle et al.’s accelerometer processing data for this phase II pilot study [61]. The Fitbit and Xiamoi Mi Band 3 step counts were used as self-monitoring tools only. They were not used in the PA outcome assessment.

#### 2.8.3. Other Patient Reported Measures

Demographics, such as age, assigned sex at birth, race/ethnicity identity, and cancer diagnosis were self-reported using paper-based questionnaires.

### 2.9. Statistical Analysis

The goal of this study was to determine the intervention’s feasibility and acceptability and to obtain exploratory pilot data to inform the design of a larger intervention study. Thus, this pilot study was not designed to have sufficient power to detect significant differences in physical function outcomes and PA. The study’s primary outcomes are feasibility and acceptability. Feasibility consisted of three components. First is the recruitment rate, second is the retention rate, and third is the group attendance rate. As previously indicated, the a priori feasibility benchmark for the recruitment rate is 0.92 participants/center/month [49], the retention rate is set at 80% or more participants completed the final study assessment [18], and group attendance is set at 75% or more attending 10 or more sessions for intervention group participants [51]. As previously indicated, the a priori acceptability benchmark is based on self-reported scores of 4 or higher for all 11 acceptability questions [19].

For assessing and comparing characteristic distributions in our samples, we used Chi-squared and Fisher’s exact test as appropriate for categorical data and *t*-tests for continuous variables. Feasibility indicators were assessed with descriptive statistics, namely frequency and percentage. The recruitment rate was calculated based on the number of participants per center per month. The retention rate was calculated as the number of total participants who completed the final assessment divided by total number of participants enrolled and randomized and multiplied by 100. The group attendance rate was calculated as the total number of participants who completed 10 or more sessions divided by the total number of participants for the intervention or control groups and multiplied by 100.

To assess secondary outcomes, we computed the difference between the last measurement and baseline for our continuous data. We report the mean of this difference, the mean baseline, and the mean of the last follow up with their standard deviations. We used these means and standard deviations to compute Cohen’s d effect size to facilitate power calculations for future studies. Data were analyzed using SPSS v 24 (IBM Corp., Armonk, NY, USA). Cohen’s d (effect size) was calculated using the effect size calculator provided by Lipsey and Wilson [62]. We took an intention-to-treat approach for study analyses and used last-observation-carried forward for missing data.

## 3. Results

### 3.1. Participants’ Characteristics

Table 2 summarizes the participants’ characteristics. Eighty percent of the participants were non-Hispanic white. The mean age was 63.75 (SD 6.35). One participant dropped out immediately after randomization, so we were not able to obtain information from the participant beyond age and race/ethnicity variables. Another participant’s baseline questionnaire was lost in the mail after the participant returned the questionnaires to the team via the USPS courier service. Multiple attempts by the team through various routes (e.g., SMS text message, email, or phone call) and at different times of the day were made (approximately 5 times on average per assessment time point) when equipment and/or questionnaires were not returned on time. We also offer to complete the questionnaire on the phone. The intent was to minimize missing data. Despite our best effort, the participant refused to complete the baseline questionnaire again. The BMI on average was 31.89 (6.04), which is considered to be in the obesity range. The majority of participants (89%) had completed active cancer treatment at baseline, and time since diagnosis averaged 96.11 months (i.e., approximately 8 years). The range was from 2 months to 284 months. No patients reported adverse events related to the intervention.

### 3.2. Feasibility and Acceptability

#### 3.2.1. Feasibility

Recruitment lasted 14 months at a single site, and the recruitment rate was 1.93 participants/center/month [49]. All 10 participants in the intervention group and eight of the 10 control group participants remained in the study and completed the final assessment (Figure 1). One participant dropped out immediately after randomization into the control group. The other control group participant dropped out right before the final assessment visit (time 2) because of caregiving demands. The mean age of the two participants who dropped out was 64 (SD 9.90), which is similar to the mean age of 18 participants who remained in the study (mean of 63.7 with SD 6.27). Both participants who dropped out were also non-Hispanic white. Among the 18 participants who remained in the study, 77.8% were non-Hispanic white. The overall retention rate was 90% (18/20). As for the group attendance rate, 88% of the intervention group participants attended 10 or more sessions, and 82% of the control group participants participated in 10 or more group calls. On average, the intervention group participants attended 11.4 sessions. As for the virtual assessments, 96% of the 56 virtual assessments were conducted without issues. Two of the 56 virtual assessments experienced internet connectivity issues. To minimize missing data collection, the team would then help to problem solve connectivity issues by turning off the assessor’s video or helped participants restart their internet router/connecting phone to a reliable WIFI hotspot.

Eight assessments were delayed (14%). Five were delayed because ActiGraphs were lost in the mail. The team was not able to recover a total of five ActiGraphs. As previously noted, the team switched to a courier service (FedEx) that provided a more reliable tracking service after the team encountered five unexpected mailing delays and loss of equipment using another mailing service. The tracking service also alerted the team to contact the research participants and remind them to complete the questionnaires and wear the ActiGraph for a minimum of 10 h a day over the next 7 days. The tracking system from FedEx also helped the team recover a few ActiGraphs that were accidentally sent to the wrong locations. Beyond loss of equipment, three assessments were delayed because either participants or their family members contracted SARS-CoV-2. We were not able to provide an ActiGraph during an active SARS-CoV-2 infection. These types of delays were not something that the team can control due to infection concerns. To minimize the lag time, the team stayed in communication with the participants on a weekly basis. The ActiGraph and questionnaires were sent out immediately as soon participants or family members recovered and were testing negative for SARS-CoV-2.

A total of two intervention group sessions experienced Zoom-related issues. Under such circumstances, we would resend the invitation links to the participants and troubleshoot on the spot to help the participants connect so they do not miss an intervention session. Overall, participants were able to follow the facilitator via SecureVideo to play the Yoga, Zuma, Kickboxing type of games on Xbox Kinect 360.

#### 3.2.2. Acceptability

**Figure 4:**
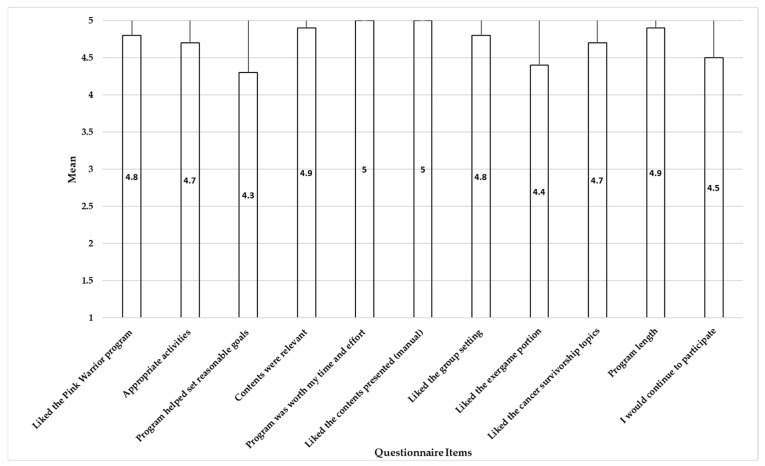
Acceptability of the Pink Warrior 2 intervention (time 2; n = 10).

Acceptance results were generated based on the 11 questions listed in Figure 4. The results indicated that overall, Pink Warrior 2, the exergame-based physical activity intervention, was acceptable. All 10 BC survivors in the intervention group rated their acceptance as 4 or higher on a 5-point scale for all 11 questions (Figure 4). Specially, they liked the exergame portion (mean of 4.4, SD of 0.84) (Figure 4; Table S2) and found that the activities were appropriate (mean of 4.7, SD of 0.68) (Figure 4; Table S2). Examples of feedback from participants after the intervention are included in the Supplemental Material (Tables S2–S4).

### 3.3. Physical Function and PA (Exploratory Results)

Table 3 shows the exploratory results of the physical function and PA assessments for the intervention and control groups. The computed mean at baseline, the mean of the last follow-up, and the mean of the differences between baseline and follow-up with their associated standard deviations by intervention and control groups are presented in Table 3. Intention-to-treat analyses included imputing one measurement using the last measured changes available carried forward for only one participant. The overall analytical sample is n = 19. However, for the TUG and two-minute step test, the overall analytical sample is n = 18. Despite having an assessment checklist and an electronic form, one of the research coordinators did not record the information for TUG and the two-minute step test for one of the participants at baseline.

Both groups started the study below the normative values of 0.99 for women 60–69 years old for gait speed [63]. The intervention group showed a greater than 1.0 point increase in SPPB score [64]. The TUG results at baseline indicated that the both groups were at increased risk for the development of disability [65]. In the 2-min step test, the intervention group started below the normative value ranges from 75 to 107 for women 60–69 years old [59]. At follow-up, the intervention group’s step counts fell within the normative range. ActiGraph data from both groups showed higher MVPA, but lower step counts were found among the intervention group participants.

## 4. Discussion

Overall, our results demonstrated that the remotely delivered exergame- and group-based PA intervention was feasible and acceptable in a group of older BC survivors, receiving treatment or not, during the COVID-19 pandemic. The study’s feasibility was demonstrated by its recruitment rate (1.93), which was above the benchmark level of 0.92 participants/center/total number of recruitment months; 90% overall retention at the end of the study; 88% adherence rate among individuals in the intervention group; minimal technological difficulties, with 96% of the 56 virtual assessments conducted without problems; and no intervention-related adverse events. The study’s acceptability was demonstrated by its mean acceptability scores, which were greater than 4 of 5 for all questions. As for the exploratory aim, both the Pink Warrior 2 intervention and attention control intervention appeared capable of producing increases in PA and function. Surprisingly, the control group participants showed an increase in steps, but the intervention group did not. Both groups had a slight increase in MVPA.

Although our enrollment met the benchmark, it is considered to be on the low end of the enrollment rate range [49]. One of the reasons for the low recruitment rate may be related to a decrease in the willingness to participate in research during the COVID-19 pandemic. Published studies indicated a lower desire to participate in research globally and a lower response rate than the pre-pandemic response rate [66,67,68]. The retention rate for the current study was at 90%, which is comparable to our previously published study [19] and is within the range (50–100%) of previous exergame-based interventions completed among cancer survivors [18]. Similar to Singh et al., a review that examined PA studies globally (e.g., USA, India, Spain, and Canada), and our previous study [19,50], the present study’s control group had a higher dropout rate than the intervention group. In fact, our intervention group’s adherence rate (88%) compared favorably to that reported by Singh et al. (81%) [50] and is within the range of retention rates (62–96.6%) of other PA interventions that included cancer survivors who were and were not currently receiving treatment [18,51].

The mean acceptability scores for all questions related to the Pink Warrior 2 intervention were ≥4, which was similar to the response to our in-person exergame- and group-based PA intervention [19]. One of the potential reasons why the Pink Warrior 2 intervention was deemed acceptable for all 11 acceptability questions was that we demonstrated modifications participants can do for the movements shown in the exergames. Similar to an exergame study completed in older adults in New Zealand, our BC survivors were able to play either standing or sitting [69]. Thus, we considered the physical abilities of various participants at each session in order to promote participants’ competence and desire to engage in doing the exergame activities based on their physical abilities, which can lead to better physical function. An additional potential reason may be related to increased enjoyment in participants when using the exergame as a tool to promote PA. Two reviews by Silva et al. [17] and Tough et al. [18] that examined the exergame interventions for persons with cancer globally (e.g., USA, South Korea, Japan, Germany, and Canada) found exergaming interventions to be more acceptable than standard of care, and they appear to improve balance, physical function, physical performance, PA levels, and reduced pain in persons with cancer. In comparison, our control group response indicated that only the appropriate activities and program length questions reach acceptability scores ≥4. The free-text responses suggested that the participants in the Pink Warrior 2 intervention in general felt connected with other participants and liked the intervention. The control group, however, did not like the survivorship navigation materials as much.

Our exploratory analysis of physical function outcomes suggested that the participants benefited from the virtually delivered exergame- and group-based PA intervention. Both intervention and control group participants were below the normative values of 0.99 m/second gait speed for women ages 60–69 years [63]. Both the intervention and control groups showed a clinically important change of ≥0.11 m/second [70]. A 0.11 m/second gait speed increase has been shown to be associated with a decreased risk in morbidity and mortality [70]. In addition, a 1.0 point increase in SPPB score was seen in the intervention group (*d* = 1.06). Brown et al. [71] observed that a 1.0 point increase in SPPB score was associated with a 12% decrease in mortality risk among survivors of cancer [71]. The TUG results at baseline (≥9 s) indicate that both intervention and control group participants were at increased risk for the development of disability [65]. However, both groups improved. The intervention group participants had a 0.69 s decrease in time, while the control group participants had a 0.01 s decrease in time (*d* = 0.43). In the 2-min step test, the intervention group started below the normative value range (75–107) but reached the normative value at the follow-up assessment [59]. We also want to highlight that the attention control group also showed improvement in physical function. This suggest the possibility of a non-specific effect of the attention control intervention.

Surprisingly, the improvements in these objectively measured outcomes for our intervention group were not matched by the group’s average step count and MVPA. This exploratory finding is in contrast with what we found in our in-person intervention, where we found an increase in steps and MVPA among intervention group participants [19]. Potential reasons for current findings may be due to SARS-CoV2–related challenges [72,73,74,75] or the use of ActiGraph among participants with slow walking speed [76]. SARS-CoV2 may have been a factor because the literature indicated a worldwide decrease in PA during the initial lockdown [72,73,74]. Additionally, Bu et al. found that despite the lifting of the initial COVID-19 lockdown, there was a steady increase in the percentage of people who continued to report not engaging in any PA [73]. Furthermore, four participants in the intervention group either had SARS-CoV2 or had family members in the same household who had SARS-CoV2. In contrast, one participant in the control group reported a household member diagnosed with SARS-CoV2. Given that recent reviews indicated that SARS-CoV2 is associated with decreases in PA and mobility [75,77], we hypothesize that this may be the underlying reason for the decrease in PA among our intervention group participants. Another potential reason for the low PA level may be related to the use of ActiGraph. Hergenroeder et al. found that ActiGraph significantly undercounted steps among individuals with slower walking speeds of <1.0 m/second [76]. This finding informs our future selection of activity monitor when designing PA interventions among individuals who may have mobility limitations.

Overall, our pilot trial had several strengths. First, it involved an innovative intervention design of delivering exergame-type activities via livestream. This was accepted by the intervention group participants, which aligns with the finding from Lin [44]. The weekly group PA sessions via the videoconference platform also did not affect the acceptability of the Pink Warrior 2 intervention compared with our in-person group findings [19]. Second, we contributed to the accumulating literature indicating that it is possible to safely conduct objective mobility, aerobic endurance, and functional fitness assessments using a videoconferencing platform [30,31]. Furthermore, supplementing the self-report measures with objective functional measure may enrich study findings, for self-reported and objective functional measures provide related but distinct information regarding an individual’s physical function [78]. Additionally, the objective functional measures may be able to capture more physical function limitations than the self-report measures [79].

Our study also had limitations related to the study design. Thus, our results need to be interpreted with caution. First, this pilot study had a small sample size and short duration. Thus, the study lacked the statistical power to detect significant differences in outcome measures and could not perform long-term monitoring of PA maintenance. We are also not able to conduct subgroup analyses to evaluate the potential effect of SARS-CoV2 in our intervention group. Second, we are not able to determine the effect of individual components of the intervention since we aimed to assess the full intervention’s feasibility and acceptability. Thus, our focus was on developing the most effective and deliverable multicomponent program remotely rather than specific intervention components. Third, our last observation carried forward has been known to underestimate effect sizes; however, our study only applied the last observation carried forward method for one individual missing one assessment. Therefore, we expect such underestimation, if any, would be minimal for our effect size estimates. Additionally, using an underestimated effect size would still result in adequate power when designing a future study. Last, the pilot trial was conducted in the southeastern Texas area. Therefore, it is limited in generalizability. However, the initial evidence will be used to inform a larger and more generalizable trial. Despite the limitations of our pilot study, we found initial evidence that our remotely delivered exergame- and group-based PA intervention was able to produce moderate to large effect size and clinically important changes on physical function, which provided initial evidence that a larger-sample trial with modifications is warranted (e.g., using a more sensitive activity monitor for populations with slower walking speed).

Our team did face several SARS-CoV2-related challenges. First, the use of a paper-based questionnaire presented a challenge. Although administrating the paper-based questionnaires was successfully implemented previously for our in-person intervention [19], we faced several logistical challenges when using paper-based questionnaires remotely during the COVID-19 pandemic. For example, there were mailing delays and a loss of questionnaire packages. Previously, when we had in-person visits, the team reviewed the questionnaires with the participants in person. Therefore, we were able to obtain missing data information immediately. Despite our efforts to contact the participants via phone, emailing the questionnaires to the participants, or offer to complete the questionnaires at assessments, parts of the four baseline questionnaires (e.g., cancer stage information or treatment information) remained missing. Thus, the team was able to obtain some of the clinical information through the electronic health record if the participant received care within the study team’s health system. With the mailing delays or loss of questionnaires, the participants were less willing to complete the full questionnaires either by phone, through emails, or at the assessment times again. Our team has since translated all questionnaires to be administered via REDCap for future studies to minimize missing data issues. Second, we also experienced losses in equipment. We were not able to recover five ActiGraphs. Third, we also experience a delay in assessment due to active SARS-CoV2 infection in the household. Overall, delays in assessments may have impacted the results, such as not having an accurate baseline and not having accurate post-intervention assessments for secondary outcomes. Our team has since changed our courier service to one with better tracking notifications to prevent further mailing delays and equipment loss. Fourth, we also experienced missing objectively collected data for one participant at baseline despite the training of coordinators, a co-development of assessment checklist, and having an electronic form. One of the coordinators was initially hired to conduct the assessments. The team did not find out about the missing data until the coordinator resigned from the position in the midst of the pandemic. Based on this lesson learned, we have since also set up documentations in the REDCap and added skip prevention to minimize missing data issues during data collection for future studies. In spite of challenges, we were able to meet the feasibility and acceptability metrics set a priori.

## 5. Conclusions

In summary, our findings lend initial evidence that a virtually delivered multicomponent PA intervention that includes exergame group play, PA behavioral coaching, and BC support is feasible and acceptable to older BC survivors regardless of their current treatment status. Additionally, our exploratory findings indicate potential physical function benefits in BC survivors, and consequently, a potential reduction in mortality of the Pink Warrior 2 intervention. However, our initial findings will need to be verified in a larger study. Using such technology can help overcome some of the limitations to PA program access experienced by older adults [37]. Additionally, we contributed to the accumulating evidence indicating that objective physical function measures can be conducted among a population with lower physical function level [30,31]. Future study is warranted to determine the effect of exergame- and group-based PA on physical function in survivors of BC. We also need to explore how to integrate the use of exergame and PA behavior coaching into cancer support groups to extend the reach of evidence-based PA programs to the wider population of cancer survivors. Lastly, our study findings can be used as initial evidence for future studies to explore its application in the clinical setting, and we can take the approach we have used in the current study to develop for use in other populations and diseases.

## Figures and Tables

**Table 1:** Details of remotely assessed objective physical function measures.

Name of the Assessment	Description	Detailed Descriptions
The Short Physical Performance Battery (SPPB)	Score range: 0 to 12 Consist of 3 components: Balance (score range from 0 to 4) Timed 10 feet walk (score range from 0 to 4) Timed 5-repeated chair stands (score range from 0 to 4)	Balance: side-by-side, semi-tandem, and tandem Timed 10 feet walk: Fastest time of 2 10 feet usual-pace walk Timed 5 repeated chair stands: repeat 5 times of rising from a chair with arms folded across the chest
Timed Up & Go TUG)	Score range: ≤10 s = normal; ≤20 s = good mobility without gait aid; ≤30 s = problems, requires gait aid; ≥14 s is associated with high fall risk	Participants need to stand up from a chair and move as quickly as participants feel safe until the participant passes a tape that is 10 feet from the chair. Then, they turn around and walk back to the chair and sit back down.
Two-minute step test	Norm for 60–64: 75–107 steps march in place for 2 min	Participants need to march in place, but the knee needs to hit the halfway mark between participants’ iliac crest and patella height.

**Table 2:** Participant characteristics.

Characteristic	Total (*N* = 20)	Intervention (*N* = 10)	Control (*N* = 10)	*p*-Value ^a^
Race/ethnicity (n = 20; n; %)				
Non-Hispanic White African American Hispanic Other	16 (80) 2 (10) 1 (5) 1 (5)	8 (80) 0 (0) 1 (10) 1 (10)	8 (80) 2 (20) 0 (0) 0 (0)	0.474
Stage (n = 18; n; %)				
0 I II III	2 (11.1) 8 (44.4) 4 (22.2) 4 (22.2)	1 (10) 2 (20) 4 (40) 3 (30)	1 (12.5) 6 (75) 0 (0) 1 (12.5)	0.106
**Treatment type** (n = 18; n; %)				
Surgery only Surgery and chemotherapy Surgery, chemotherapy, and radiation Surgery and radiation	1 (5.6) 4 (22.2) 8 (44.4) 5 (27.8)	1 (10) 2 (20) 5 (50) 2 (20)	0 (0) 2 (25) 3 (37.5) 3 (37.5)	0.904
**Current treatment status** (n = 18; n; %)				
Off treatment On treatment	16 (89) 2 (11)	8 (80) 2 (20)	8 (100) 0 (36.67)	0.477
**Patient-reported neuropathy** (n = 18; n; %)				
Yes No	6 (33.3) 12 (66.7)	4 (40) 6 (60)	2 (25) 6 (75)	0.638
**Age** (n = 20; years, range 55–79; mean; SD)	63.75 (6.35)	64.90 (8.03)	62.60 (4.20)	0.43
**Time since diagnosis** (n = 18; months; mean; SD)	96.11 (82.61) Range: 2–284 months	113.70 (92.99)	74.13 (66.81)	0.33
**BMI** (n = 19; kg/m^2^; mean; SD)	31.89 (6.04)	33.91 (7.11)	29.66 (3.80)	0.13

^a^ *p*-values calculated using Fisher’s exact test for categorical variables and the two-sample *t*-test for continuous variables.

**Table 3:** Differences between intervention and control groups (intention-to-treat analysis).

Variables	Intervention			Control			Effect Size
	Baseline Mean (SD)	Follow-Up Mean (SD)	Mean of Difference (SD)	Baseline Mean (SD)	Follow-Up Mean (SD)	Mean of Difference (SD)	Cohen’s d
Gait speed (meter/seconds); n = 19	0.76 (0.24)	0.94 (0.17)	0.18 (0.17)	0.89 (0.18)	1.01 (0.15)	0.11 (0.13)	0.46
Total SPPB ^a^ score; n = 19	8.70 (1.57)	10.30 (1.34)	1.6 (1.17)	9.56 (1.59)	10 (1.12)	0.44 (1.01)	1.06
TUG ^b^ (seconds); n = 18	10.46 (3.52)	9.78 (3.11)	−0.69 (0.91)	9.12 (1.73)	8.93 (0.85)	−0.01 (2.06)	0.43
Two-minute step test (count); n = 18	62.89 (21.69)	75.0 (24.26)	12.11 (13.83)	75.89 (30.98)	76.11 (28.81)	0.22 (24.11)	0.61
Steps (average steps); n = 19	4652.60 (2659.88)	4423.09 (2016.41)	−229.52 (1905.94)	4268.52 (1721.36)	5838.69 (2767.52)	1570.17 (2355.59)	0.85
MVPA ^c^ (average minutes); n = 19	9.4689 (9.93)	10.00 (9.13)	0.54 (8.78)	12.07 (13.67)	17.34 (23.09)	5.28 (23.66)	0.27

^a^ SPPB: Short Physical Performance Battery; ^b^ TUG: Timed Up & Go; ^c^ moderate-vigorous physical activity.

## Data Availability

Requests for data may be sent to the corresponding author. Data would be made available following the University of Texas Medical Branch Data Sharing Policy.

## Supplementary Materials

The following supporting information can be downloaded at: https://www.mdpi.com/article/10.3390/life13020574/s1, Table S1: Detailed descriptions of how the SPPB, TUG, and 2-min step test were conducted virtually; Table S2: Participants’ written feedback regarding the Pink Warrior 2 and the support programs; Table S3: Acceptability of the Pink Warrior 2 intervention (time 2; n = 10); Table S4: Acceptability of the UTMB support group program (time 2; n = 7); Table S5: Consort 2010 checklist of information for reporting a pilot or feasibility trial.
