# Supplementary material for: Assessing the Suitability of a Virtual ‘Pink Warrior’ for Older Breast Cancer Survivors during COVID-19: A Pilot Study
Source: Life (Basel). 2023 Feb 18;13(2):574. doi: 10.3390/life13020574 (PMC9965453; doi:10.3390/life13020574)
Supplement: Supplementary file 1 [file life-13-00574-s001.zip › life-2178123-supplementary.pdf]

Supplementary Materials

Table S1. Detailed descriptions of how the SPPB, TUG, and 2-minute step test were conducted virtually

**Test and Directions on how to conduct the remote assessments**
**Short Physical Performance Battery**<br>*Tools:*<br>-10 feet steel tape measure<br>-Colored painter's tape<br>-Smartphone and phone stand<br>*Instructions:*<br>Follow the script provided as part of the geriatric toolkit (https://geriatrictoolkit.missouri.edu/SPPB-Score-Tool.pdf) and https://sppbguide.com/. The participant was asked to measure a distance of 10 feet for the gait speed test and to mark the starting and stopping points on the floor with colored painter's tape. The assessor demonstrated each movement, asked the participant to demonstrate a practice run, and asked the participant to adjust their camera angle as needed prior to beginning each SPPB test.
**Timed Up & Go**<br>*Tool:*<br>-10 feet steel tape measure<br>-A stable chair<br>-Colored painter's tape<br>-Smartphone and phone stand<br>*Instructions:*<br>Follow the script provided as part of the CDC's STEADI kit (https://www.cdc.gov/steadi/pdf/TUG_test-print.pdf). The assessor demonstrated the movement, asked the participant to demonstrate a practice run, and asked the participant to adjust their camera angle as needed prior to beginning the test.
**Two-minute step test**<br>*Tools:*<br>-10 feet Steel tape measure<br>-A stable chair without wheels<br>-Colored painter's tape<br>-Smartphone and phone stand<br>*Instructions:*<br>1. The assessor and the participant work on the camera angle so the assessor can see the wall and the participant.<br>2. Stand sideways next to the wall (see the picture below).<br>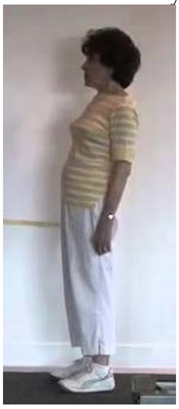<br>3. Hold the bottom of the tape measure against the top of the hip bone (iliac crest), then extend the tape to the wall and put a horizontal mark on the wall with the color tape (see the picture below).

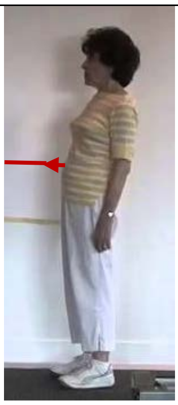

4. Then the participant would stand straight up next to the wall with their hip (right/left) touching the wall (see the picture below).

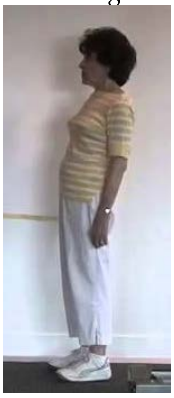

5. The participant would then bend at the waist but keep the knee that is closest to the wall straight and then extend the steel tape from the kneecap (patella) to the wall. They then make a horizontal tape mark on the wall.
6. Then they measure the distance between the two tape marks and put a horizontal mark midway between the two marks. The top and bottom pieces of tape were then removed to avoid assessor confusion, leaving only the middle piece of tape marking the distance halfway between the iliac crest and the patella.
7. The assessor would then ask the participant to stand next to the wall (see the picture in #3) and raise the knee to the mark as a practice run.
8. The assessor would then mark this spot on her screen with her mouse cursor or a small Post-it note in case the tape became obstructed from view by the participant unknowingly moving in front of it during the test.
9. Then begin test.

Table S2. Participants' written feedback regarding the *Pink Warrior 2* and the support programs.

**What I like the most about the Pink Warrior program (intervention group)**
"Meeting with people and hearing different ideas." (PW205)
"Meeting with others who understood my diagnosis and side effects. I also appreciated links to exercise videos to do on my own." (PW213)
"The weekly meetings, I looked forward to them." (PW221)
"It was conducive to time restraints and flexible when needed. Instructors were pleasant and encouraging." (PW211)
"reminded me to put my health first." (PW207)
**What I like the least about the Pink Warrior program (intervention group)**
"Paperwork…" (PW211)
"Filling out this [questionnaire]." (PW214)
"The slow exercises—yoga. I liked more high energy things." (PW205)
"these forms." (PW221)
"nothing." (PW227)
**What I like the most about the support program (control group)**
"The discussion of the other ladies on how they are handling life after cancer." (PW202)
"Just another chance to talk to real people during the pandemic when I was not able to get out and do as I normally would." (PW210)
"Our moderator was empathetic and supportive." (PW212)
"Opportunity to share feelings, ask questions and learn from others." (PW219)
"Was the fact a relationship was established." (PW225)
**What I like the least about the support program (control group)**
"No improvement needed." (PW203)
"I am further out. The material only applied in retrospect." (PW206)
"The group primarily was used as a way to force me to exercise more." (PW210)
"I never felt connected with the people in the group." (PW212)
"We could not meet in person, but understood it was best to do Zoom" (PW225)

Table S3. Acceptability of the Pink Warrior 2 intervention (time 2; n=10)

Items	Mean (SD)
Liked the Pink Warrior program	4.8 (0.42)
Appropriate activities	4.7 (0.68)
Program helped set reasonable goals	4.3 (0.95)
Contents were relevant	4.9 (0.32)
Program was worth my time and effort	5.0 (0.0)
Liked the contents presented (manual)	5.0 (0.0)
Liked the group setting	4.8 (0.63)
Liked the exergame portion	4.4 (0.84)
Liked the cancer survivorship topics	4.7 (0.68)
Program length	4.9 (0.32)
I would continue to participate	4.5 (1.27)

Table S4. Acceptability of the UTMB support group program (time 2; n=7)

Items	Mean (SD)
Liked the support group	3.71 (1.38)
Appropriate activities	4.00 (1.41)
Program helped set reasonable goals	3.71 (1.38)
Contents were relevant	3.43 (1.51)
Program was worth my time and effort	3.71 (1.38)
Liked the contents presented (manual)	3.57 (1.40)

Liked the group setting	3.14 (1.77)
Program length	4.00 (1.41)
Intend to tell others about the support group	3.71 (1.50)
I would continue to participate	3.57 (1.40)

Table S5. Consort 2010 checklist of information for reporting a pilot or feasibility trial

Section/Topic	Item No	Checklist item	Reported on page No
**Title and abstract**			
	1a	Identification as a pilot or feasibility randomised trial in the title	1
	1b	Structured summary of pilot trial design, methods, results, and conclusions (for specific guidance see CONSORT abstract extension for pilot trials)	1
**Introduction**			
Background and objectives	2a	Scientific background and explanation of rationale for future definitive trial, and reasons for randomised pilot trial	2,3
	2b	Specific objectives or research questions for pilot trial	2,3
**Methods**			
Trial design	3a	Description of pilot trial design (such as parallel, factorial) including allocation ratio	3
	3b	Important changes to methods after pilot trial commencement (such as eligibility criteria), with reasons	N/A
Participants	4a	Eligibility criteria for participants	3
	4b	Settings and locations where the data were collected	3
	4c	How participants were identified and consented	3
Interventions	5	The interventions for each group with sufficient details to allow replication, including how and when they were actually administered	4-7
Outcomes	6a	Completely defined prespecified assessments or measurements to address each pilot trial objective specified in 2b, including how and when they were assessed	7-9
	6b	Any changes to pilot trial assessments or measurements after the pilot trial commenced, with reasons	N/A
	6c	If applicable, prespecified criteria used to judge whether, or how, to proceed with future definitive trial	7
Sample size	7a	Rationale for numbers in the pilot trial	8
	7b	When applicable, explanation of any interim analyses and stopping guidelines	N/A
Randomisation:			
Sequence generation	8a	Method used to generate the random allocation sequence	4
	8b	Type of randomisation(s); details of any restriction (such as blocking and block size)	4
Allocation concealment mechanism	9	Mechanism used to implement the random allocation sequence (such as sequentially numbered containers), describing any steps taken to conceal the sequence until interventions were assigned	4

Implementation	10	Who generated the random allocation sequence, who enrolled participants, and who assigned participants to interventions	4
Blinding	11a	If done, who was blinded after assignment to interventions (for example, participants, care providers, those assessing outcomes) and how	4
	11b	If relevant, description of the similarity of interventions	N/A
Statistical methods	12	Methods used to address each pilot trial objective whether qualitative or quantitative	8,9
**Results**			
Participant flow (a diagram is strongly recommended)	13a	For each group, the numbers of participants who were approached and/or assessed for eligibility, randomly assigned, received intended treatment, and were assessed for each objective	9
	13b	For each group, losses and exclusions after randomisation, together with reasons	3,9
N/	14a	Dates defining the periods of recruitment and follow-up	3-5, 10
	14b	Why the pilot trial ended or was stopped	N/A
Baseline data	15	A table showing baseline demographic and clinical characteristics for each group	9,10
Numbers analysed	16	For each objective, number of participants (denominator) included in each analysis. If relevant, these numbers should be by randomised group	10,11
Outcomes and estimation	17	For each objective, results including expressions of uncertainty (such as 95% confidence interval) for any estimates. If relevant, these results should be by randomised group	10, 11
Ancillary analyses	18	Results of any other analyses performed that could be used to inform the future definitive trial	10,11
Harms	19	All important harms or unintended effects in each group (for specific guidance see CONSORT for harms)	N/A
	19a	If relevant, other important unintended consequences	N/A
**Discussion**			
Limitations	20	Pilot trial limitations, addressing sources of potential bias and remaining uncertainty about feasibility	13
Generalisability	21	Generalisability (applicability) of pilot trial methods and findings to future definitive trial and other studies	13
Interpretation	22	Interpretation consistent with pilot trial objectives and findings, balancing potential benefits and harms, and considering other relevant evidence	11-13
	22a	Implications for progression from pilot to future definitive trial, including any proposed amendments	11-13
**Other information**			
Registration	23	Registration number for pilot trial and name of trial registry	5
Protocol	24	Where the pilot trial protocol can be accessed, if available	5
Funding	25	Sources of funding and other support (such as supply of drugs), role of funders	14
	26	Ethical approval or approval by research review committee, confirmed with reference number	5, 14

Adapted from the template by Eldridge SM, Chan CL, Campbell MJ, Bond CM, Hopewell S, Thabane L, et al. CONSORT 2010 statement: extension to randomised pilot and feasibility trials. BMJ. 2016;355.
